# The Effect of Static and Dynamic Visual Stimulations on Error Detection Based on Error-Evoked Brain Responses

**DOI:** 10.3390/s20164475

**Published:** 2020-08-10

**Authors:** Rui Xu, Yaoyao Wang, Xianle Shi, Ningning Wang, Dong Ming

**Affiliations:** 1 Academy of Medical Engineering and Translational Medicine, Tianjin University, Tianjin 300072, China; xrblue@tju.edu.cn (R.X.); wangyaoyao@tju.edu.cn (Y.W.); 2 Department of Biomedical Engineering, College of Precision Instruments and Optoelectronics Engineering, Tianjin University, Tianjin 300072, China; xl_s@tju.edu.cn (X.S.); wnn_148@tju.edu.cn (N.W.)

**Keywords:** error-related potentials (ErrPs), static visual stimulation, dynamic visual stimulation, time window, error detection

## Abstract

Error-related potentials (ErrPs) have provided technical support for the brain-computer interface. However, different visual stimulations may affect the ErrPs, and furthermore, affect the error recognition based on ErrPs. Therefore, the study aimed to investigate how people respond to different visual stimulations (static and dynamic) and find the best time window for different stimulation. Nineteen participants were recruited in the ErrPs-based tasks with static and dynamic visual stimulations. Five ErrPs were statistically compared, and the classification accuracies were obtained through linear discriminant analysis (LDA) with nine different time windows. The results showed that the P3, N6, and P8 with correctness were significantly different from those with error in both stimulations, while N1 only existed in static. The differences between dynamic and static errors existed in N1 and P2. The highest accuracy was obtained in the time window related to N1, P3, N6, and P8 for the static condition, and in the time window related to P3, N6, and P8 for the dynamic. In conclusion, the early components of ErrPs may be affected by stimulation modes, and the late components are more sensitive to errors. The error recognition with static stimulation requires information from the entire epoch, while the late windows should be focused more within the dynamic case.

## 1. Introduction

When people recognize the occurrence of an error, potentials are evoked in the brain. They are the error-related potentials (ErrPs) [1]. Generally, the ErrPs can reflect the respond of the brain to errors. Previous studies with source localization suggest that the ErrPs are generated in the anterior cingulate cortex (ACC) after errors occur [2,3]. The components of ErrPs are reported to be the most obvious potentials at frontocentral sites (FCz) [4].

ErrPs are commonly used for error detection and performance monitoring in brain-computer interface (BCI). Sometimes the BCI decodes the brain signals incorrectly and makes a decision that does not match the user’s intention. In this condition, ErrPs provide a new idea to improve the BCI’s performance [5]. Kalaganis et al. [6] found that ErrPs detection was a useful remedy to coping with typesetting errors in the use of gaze-based keyboards. Yousefi et al. [7] attempted to design a BCI system based on non-motor imagery (MI) mental tasks and randomly chose a subset of 20% trials with incorrect feedback. The results confirmed the presence of interaction ErrPs, and after applying ErrPs-based error correction, the classification accuracies of the two tasks increased by 9% and 14%, respectively.

Observation ErrPs are the key point of error recognition in BCI control. They are generated when a person observes an error in the task performed by a second subject (an agent or other human) [8,9]. The observation ErrPs are usually evoked by visual stimulations, which can be roughly divided into static and dynamic ones. The static stimulations mostly use static symbols, letters or numbers as the stimulus, such as those in the Eriksen flanker task [3] and Go/NoGo task [10]. In BCI control, the P300-spellers are the common static visual stimulations. Fernandez-Rodriguez et al. [11] designed the P300-spellers employing the letters and block-shaped figures to study the impact of these two figures on classification performance. Compared with the static stimulations of ErrPs, the participants receiving the dynamic stimulations will observe the dynamic trajectory, such as the target moving task with interference [12,13] and the cursor control task [14]. Zander et al. [15] proposed to control the cursor with moving cursor-evoked ErrPs, and finally realized the neural adaptability of the BCI system. In the human-robot interactions, dynamic stimulations are more commonly applied [16], e.g., execution of trajectories in an end effector reaching task [17] or association of objects in a sorting task [18], as well as recognition and imitation of human gestures [19]. Consequently, both static and dynamic stimulations are widely used in BCI control, and both the static and dynamic errors are common.

In order to improve the error recognition rate based on ErrPs, previous studies have tried error-related features with different time windows. Certainly, some studies directly selected the whole epoch to extract classification features [5], and some chose the most suitable time window for better classification. Ventouras et al. [20] adopted the Eriksen flanker tasks and three time windows to classify correct from incorrect actions. Finally, they got a better classification through the earlier time window, including only the error-related negativity, compared to the other two time windows (which also included error positivity or the whole epoch). Kakkos et al. [21] detected correct and incorrect responses to pictures and audios, based on waveform characteristics of ErrPs form different time windows. They first divided four time windows (tw1, tw2, tw3, and tw4) according to the obtained waveforms, and then got the two-window combinations: tw1 and tw2 (tw1,2), tw2 and tw3 (tw2,3), and tw3 and tw4 (tw3,4). The result showed that the combination of two time windows got better performance with accuracies larger than 80%. In a word, the selection of the time windows is very important, and different studies with different stimulations choose different time windows to achieve the best classification performance. Therefore, we hypothesized that different visual stimuli (static and dynamic) may affect the selection of time windows, and further affect the recognition of errors.

The aims of this study are to find the difference in ErrPs’ characteristics of different visual stimulation modes (static and dynamic), and figure out the influence of stimulation mode on time window selection. Therefore, we designed an experiment to evoke ErrPs with static and dynamic visual stimulations. Five electroencephalogram (EEG) components (N1, P2, P3, N6, and P8, named by the occurrence times) were calculated and analyzed to investigate the difference in ErrPs between static and dynamic stimulations. Then, we chose nine different time windows for classification recognition. Based on ErrPs’ five components, we got five time windows. The other two time windows were the combined windows selected based on the results of waveform analysis. The last two time windows were obtained by dividing the whole epoch into two segments. Finally, we employed the linear discriminant analysis (LDA) classifier to classify the correctness and error. We expected to find the difference between static and dynamic stimulations and the influence of the stimulation modes on the selection of time windows for the improvement of BCI performance.

## 2. Materials and Methods

### 2.1. Participants

Nineteen healthy participants (8 females and 11 males; mean age: 20.7 ± 2.34 years old) took part in the study. They had no history of neurological or psychiatric disease. All participants signed informed consent before the experiment. The study was approved by the ethics committee of Tianjin University.

### 2.2. Experiments

**Figure 1:**
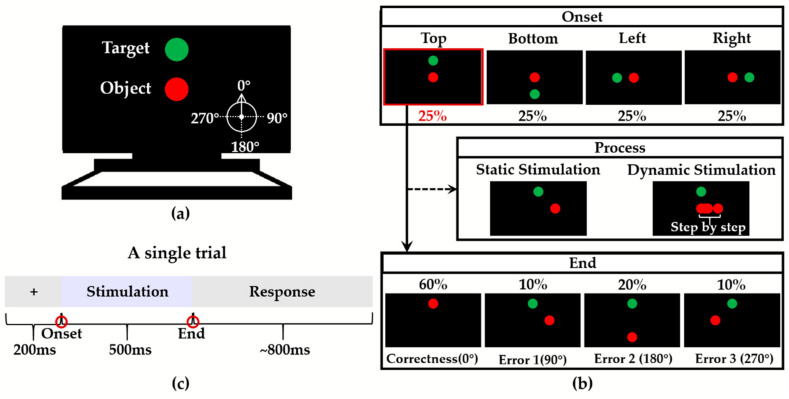
(**a**) Screen display at the onset of the experiment (the correct position: top); (**b**) Design of correct and incorrect events in this experiment (the picture showed the green ball appearing the top) and illustration of the onset, process and end of stimulations; (**c**) Setup of a single trial in the experiment.

In this experiment, participants would see two balls (Figure 1a) on the screen: A green ball indicated the target direction, and a red ball indicated the object that the participant needed to focus on. At the onset of each trial, a red ball appeared in the center of the screen. At the same time, the green ball appeared randomly at one of the four positions (top, bottom, left and right of the center of the screen, each with a probability of 25%; equal distance between the red and green balls at the onset for different trials) (Figure 1b). The correct events were when the red ball moved toward the green ball with 60% probability. The incorrect events were when the red ball did not move toward the green ball (that were the other three directions). The error 1 was a 90° clockwise position relative to the correct position with 10% probability. The error 2 was a 180° clockwise position relative to the correct position with 20% probability. The error 3 was a 270° clockwise position relative to the correct position with 10% probability.

The experiment consisted of two parts, which were the static and dynamic visual stimulation tasks (Figure 1b). In the static visual stimulation task, the red ball disappeared from the starting position (center of the screen) and appeared at the final position (one of the top, bottom, left or right). The stimulation process of the dynamic visual stimulation task was: After the stimulation started, the red ball moved randomly in a certain direction (uniform speed), and reached the final position after 500 ms. Moreover, in the static tasks, participants accepted the pictures of the beginning and end of the dynamic tasks.

A single trial contained 200 ms of the preparation, 500 ms of the stimulation, and approximate 800 ms of the response (Figure 1c). Participants needed to make judgments after the stimulation was over. There were four blocks in the experiment with each task containing two blocks. Participants had enough rest time between every two blocks. Each block had 240 trials (correctness: 144 trials; errors: 96 trials). Before the formal experiment, participants needed to practice the paradigm.

### 2.3. Data Recording and Pre-Processing

The EEG signals were acquired by a commercial device (NeuroScan, SynAmps2/SCAN, Charlotte, NC, USA), hardware-filtered in the frequency range of 0.015–250 Hz and sampled at 1000 Hz. EEG data were recorded with 64 electrodes located in the positions following the 10/20 system. The recorded data were referenced to the right mastoid and grounded at the prefrontal lobe. An additional 50 Hz notch filter was employed during data acquisition.

Data analyses were performed using Matlab R2014b (MathWorks, Natick, MA, USA), with the toolbox EEGLAB (Swartz Center for Computational Neuroscience, LA Jolla, CA, USA; https://sccn.ucsd.edu/eeglab/index.php). Because of the visual stimuli used in this experiment, participants would produce eye movements that affect EEG signals, such as blinking. Even if participants were told to reduce eye movements and keep still before the experiment, eye movements and excessive muscle activities could not be completely avoided. In that way, the components related to these disturbances were identified by visual inspection and removed after independent component analysis (ICA). The acquired EEG data were re-referenced to the average of the two mastoid and filtered (0.01 ~ 12 Hz) by a 4th-order zero-phase Butterworth filter [22]. In all cases, epochs were rejected according to ±1000 µV threshold criteria for removing severe body shaking. Then the EEG was down-sampled to 500 Hz. Because the interval of each trial was set to about 1500 ms, data were segmented into the epoch from 200 ms pre-stimulus to 1000 ms post-stimulus, and the baseline was corrected to a 100 ms epoch from −100 to 0 ms before the stimulation onset. Finally, four grand average EEG waveforms were obtained from all trials of all participants, which were static correctness and error EEG and dynamic correctness and error EEG.

### 2.4. Data Processing and Analysis

**Figure 2:**
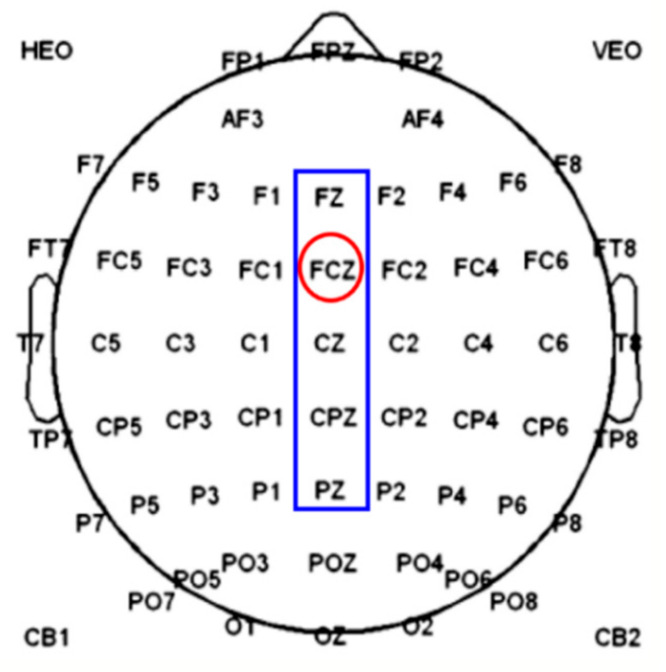
The location of five selected channels. FCz channel (inside the red circle) was used for error-related potentials (ErrPs) waveform analysis, and the five channels (inside the blue box) were used for classification. FCz: Frontal central; HEO: Horizontal electrooculogram; VEO: Vertical electrooculogram.

The EEG data at the electrode FCz (Figure 2) was analyzed in this study. We obtained the average EEG waveforms of the correctness and error, under the static and dynamic visual stimulations. There were five components chosen and calculated, i.e., N1, P2, P3, N6, and P8 (Table 1).

In this study, we analyzed the components that were sensitive to errors in static and dynamic conditions and the difference between static and dynamic errors. All the components mentioned above were compared between correctness and error, or between static and dynamic errors with Wilcoxom signed rank test.

### 2.5. Classification

**Figure 3:**
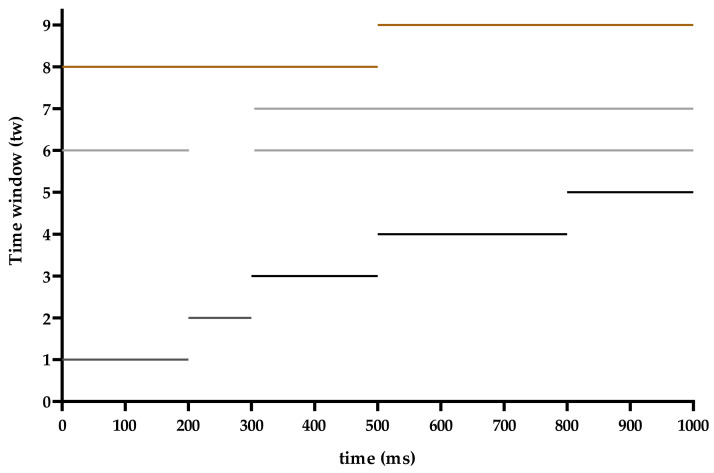
The time interval corresponding to Time windows (tw1–9). The x-axis represented time selection. The y-axis represented tw1–9.

Through the analysis of waveform characteristics, we could obtain the sensitivity to errors under the static and dynamic stimulations, and the difference between static and dynamic stimulations in error cases. In order to further demonstrate the impact of the difference between static and dynamic stimulations on the classification performance of BCI, we chose nine different time windows (tw1–9) (Figure 3). The time windows were obtained from three cases. Firstly, according to the occurrence time of ErrPs’ five components (N1, P2, P3, N6, P8), tw1–5 were selected. Secondly, two combined windows (tw6 and tw7) were related to the occurrence time of the sensitive components to errors under static and dynamic stimulations, respectively. Among them, tw6 was corresponded to N1, P3, N6, P8, and tw7 was corresponded to P3, N6, P8. Therefore, tw1–7 were all related to the five components, where tw1–5 were corresponding to five components (N1, P2, P3, N6, and P8) respectively, and tw6 and tw7 were the combination of tw1–5 according to error-sensitive components in static and dynamic conditions. Finally, to observe the nature of static and dynamic error recognition on time window, the whole epoch was divided into two segments: Tw8 from 0 to 500 ms and tw9 from 500 to 1000 ms. In terms of time, tw8 contained tw1–3, and tw9 contained tw4–5.

We first down-sampled the data to 25 Hz for each trial continuedly, and the data samples of the five channels (Fz, FCz, Cz, CPz, Pz) at the cortex midline (Figure 2) within different time windows (tw1 to tw9) were used for classification. The down-sampled data in one individual trial was fed to the classification as one sample. The ninety percent of the trials of each participant were used as the training set, and the remaining 10% were used as the test set. LDA classifier and ten-fold cross-validation were adopted for classification recognition.

After classification, two-way repeated-measures analysis of variance (ANOVA) was submitted for accuracy rates, with stimulation (static, dynamic) and time window (tw1–9) as the within-subject factors. If the interaction effect of the two factors was significant, the simple effect analysis was performed. The simple effect refers to the difference between different levels of the same factor, with the level of other the factor fixed.

## 3. Results

### 3.1. Components of Error-Related Potentials

**Figure 4:**
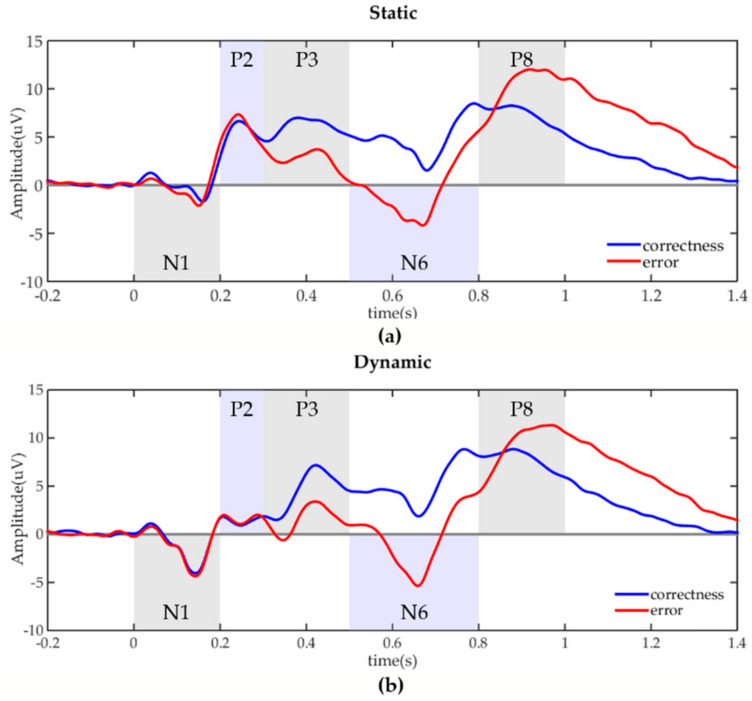
The grand average electroencephalogram (EEG) of all subjects at FCz channel. (**a**) Static visual stimulation; (**b**) Dynamic visual stimulation.

Figure 4 showed the EEG waveforms evoked by correctness and error under the static and dynamic visual stimulations, respectively. It was revealed that the errors in these two cases both evoked lower P3, and N6 compared to the correctness, and the error-related positive component P8 at around 800 ms appeared to increase. There was a definite peak for static stimulation, while several small peaks for dynamic stimulations between 200 ms to 300 ms after the stimulus. Table 2 contained the calculated components (N1, P2, P3, N6, P8) and the comparison result of these components between correctness and error. P3, N6, and P8 were significantly changed for correctness and error under both static and dynamic stimulations. There was a significant variation of N1 only in static tasks, but not in dynamic tasks.

**Figure 5:**
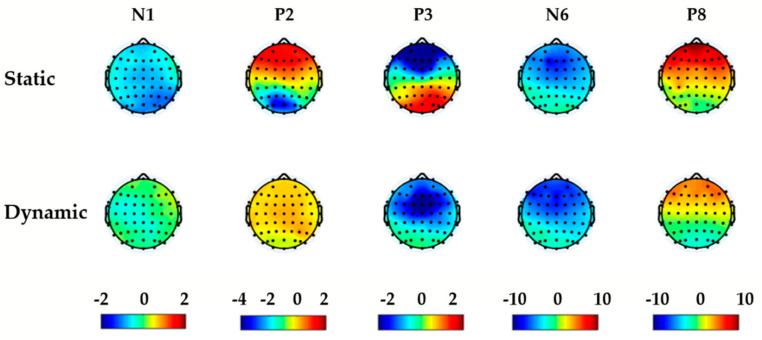
Scalp topographical maps of the difference values between error and correctness (error minus correctness) under static and dynamic stimulations, for each component (N1, P2, P3, N6, P8).

Figure 5 showed the scalp topographical maps of the difference values between error and correctness under static and dynamic stimulations. The difference values were obtained by error minus correctness of five components. The maximal differences of N1, P2, P3, and N6 appeared at the front-central electrode sites.

**Figure 6:**
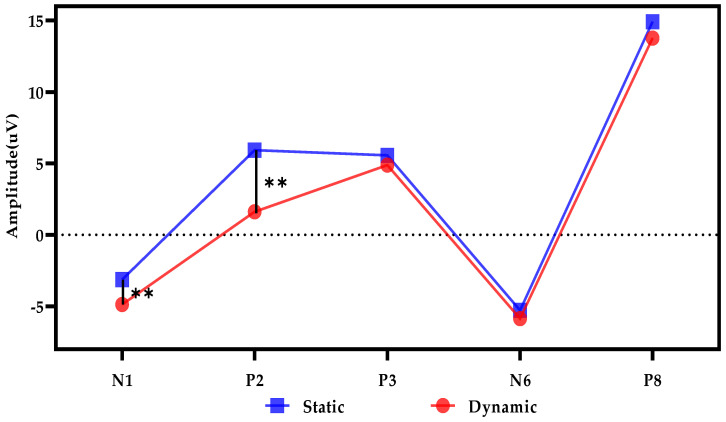
The N1, P2, P3, N6, and P8 amplitudes of the error case under static and dynamic visual stimulations. **: *p* ≤ 0.01.

In order to find the difference between the two stimulation modes, it was also necessary to directly compare the EEG in the error case. Figure 6 showed the values of the five components of the error cases under static and dynamic stimulations. The components in dynamic stimulation were always smaller than those in static cases. In other words, the amplitudes of P2, P3, and P8 were larger in the static case; the amplitudes of N1 and N6 were larger in the dynamic case. There were significant differences in N1 and P2 between static and dynamic (N1: *p* < 0.01; P2: *p* < 0.01), but there was no difference in P3, N6, and P8. Generally, the distinction between the two stimulation modes occurred within about 300 ms after the start of stimulation.

### 3.2. Classification Accuracies of Different Visual Stimulation Modes and Different Time Windows

**Figure 7:**
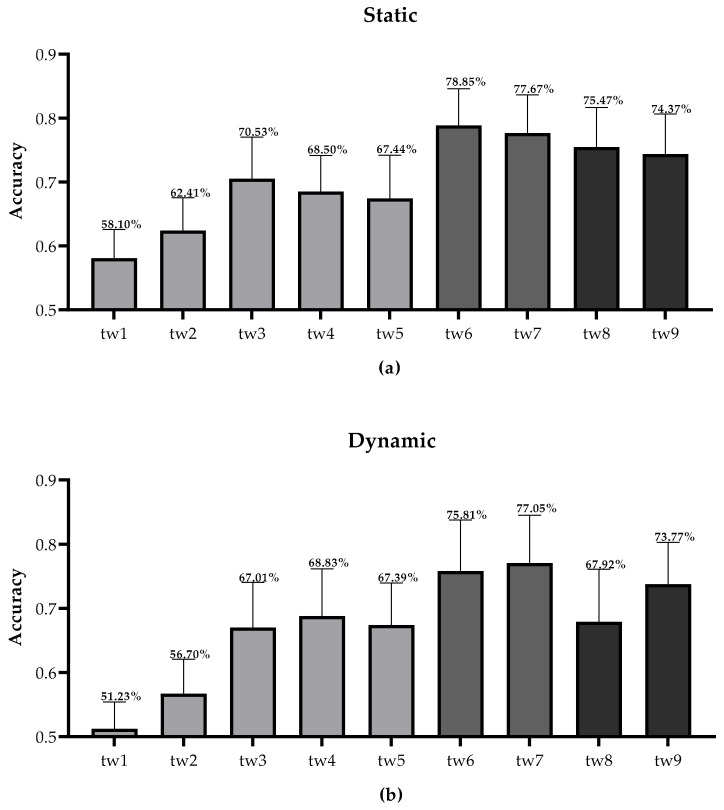
The classification accuracies of different time windows (tw1–9) under two visual stimuli. (**a**) Static visual stimulation. (**b**) Dynamic visual stimulation.

Figure 7 showed the classification accuracy results of different time windows under static and dynamic stimulations. Tw1–5 corresponded to the time selection of N1, P2, P3, N6, and P8, respectively. Similar to the waveform components analysis, tw3, tw4, and tw5 were all larger than the recognition accuracies of tw1 and tw2. Differently, tw1 (corresponding to N1) in the static condition did not achieve a good recognition effect. Consistent with the result of components analysis, the tw6 of the static condition (corresponding to N1, P3, N6, and P8) and the tw7 of the dynamic condition (corresponding to P3, N6, and P8) achieved the best classification accuracy. The classification accuracy of tw8 was higher than tw9 under static stimulation, but tw9 was higher than tw8 under dynamic stimulation. This indicated that the accuracy of the static condition was higher in the first 500 ms after the start of stimulation, while the accuracy of dynamic condition was higher after 500 ms.

In order to investigate whether there were significant differences between the classification accuracy results, repeated-measures ANOVA was performed for accuracies. The results showed that the main effects of stimulation and time window were significant (stimulations: F(1,18) = 13.505, *p* = 0.002; time windows: F(4.146,74.634) = 104.528, *p* = 0.000), and the interaction between stimulation and time window was significant (F(3.860,69.484) = 9.257, *p* = 0.000). This meant that both stimulation and time window could affect the classification accuracy.

As there was a significant interaction between the simulation mode and the time window, the simple effect of each factor was performed. Firstly, the difference between the two stimulation modes for each time window was inspected. Tw1, tw2, tw3, tw7, and tw8 displayed significant differences between static and dynamic stimulations (Table 3). Secondly, the differences among different time windows were inspected. At both static and dynamic stimulation modes, the accuracies with different time windows were significantly different (static: F(4.396,79.121) = 70.775, *p* = 0.000; dynamic: F(3.812,68.622) = 78.295, *p* = 0.000). The results of the pairwise comparisons were listed in Table 4. On the one hand, tw1–5 reflected the differences between different EEG components. The accuracies with tw3, tw4, and tw5 were significantly higher than those with tw1 and tw2 under both static and dynamic stimulations. On the other hand, tw6–9 were analyzed to find the best time window for different visual stimulation modes. At the static level, tw6, achieving the highest accuracy, showed significant differences from tw8 and tw9, respectively; at the dynamic level, tw7 with the highest accuracy, showed significant differences from tw8 and tw9, respectively. Moreover, the accuracy of tw9 was significantly higher than tw8 under dynamic stimulation.

## 4. Discussion

We have obtained results about the impact of different visual stimulations (static or dynamic) on error recognition. In the EEG analysis, P3, N6, and P8 could well reflect the differences between correctness and error under both static and dynamic stimulations, while N1 only made sense under static stimulation. By comparing the static and dynamic error EEG, N1 and P2 showed significant differences. In classification recognition, both stimulation mode and time window affected the accuracies. In static and dynamic conditions, the accuracies with tw3, tw4, tw5 were significantly higher than those with tw1 and tw2. The highest accuracy occurred with tw6 in static tasks, and with tw7 in dynamic tasks. Moreover, the error recognition based on ErrPs of the late time window was better in dynamic stimulation mode. Static and dynamic visual stimuli in ErrPs-based BCI systems have been widely used, e.g., P300-BCIs with ErrPs correction to improve accuracies [9,23], human-robot co-adaptation using ErrPs [16], and implicit cursor control based on ErrPs [15]. This paper studied the difference between the two stimulation modes and the selection of time window, to improve the BCIs’ error recognition accuracy.

### 4.1. EEG Components about the Difference between Correctness and Error

The early components of this study were N1 and P2, of which only N1 showed sensitivity to error under static condition. In existing choice reaction tasks, the error-related negativity (ERN) or error negativity (Ne) at around 50–150 ms after an erroneous response was focused on responding to errors [24]. Depending on the task, an error-related positive peak, called as error positivity (Pe), follows the ERN. Moreover, most tasks using ERN employed static visual stimulations to explore neural mechanisms [25,26]. White et al. [25] adopt the Eriksen flanker tasks examining the influence of task-irrelevant uncertain (related to anxiety) evaluative threat on error-monitoring using ERN. Thus, the static N1 was like ERN. However, this study did not observe the Pe-likely component. Only under static stimulus, a clear positive peak P2 appeared, which is possible because the static visual stimulus was simpler and more stable than the dynamic stimulus [27].

Distinguished from the odd-ball paradigm, the correct and incorrect trials were randomly arranged, and the correct direction also varied with the trials randomly. The result was that the correctness trials with a greater probability (60%) caused a larger P3 than error trials under these two stimulus conditions. This was opposite to the result of the odd-ball task: the smaller the probability of target occurrence, the greater the amplitude of P300 (P3) [28]. Studies have shown that P300 was also related to the satisfaction of expectations [29]. Lei et al. [30] found a related-category condition elicits larger P300 relative to an unrelated-category condition, and demonstrated that greater expectation satisfaction is associated with larger P300 amplitude. In this study, participants expected the correct events to happen more, while the errors deviated from their expectations. Similarly, we got a larger P3 in the more anticipated correct events.

There were two late components, N6 and P8, which also showed significant differences between correctness and error. Kouider et al. [31] argued that the late slow wave (LSW) starting at 700 ms and peaking at 1000 ms may reflect a conscious response to violate the expectation in their task, because invalid cues caused lager negative LSW. Positive slow wave (PSW) has been shown to be related to the evaluation of the correctness [32,33]. Stuss et al. [32] got two PSW which were both of greater amplitude in the trials before the subject finally confirmed the correct criterion. Thus, our N6 and P8 can also serve as indexes of the evaluation of correctness or errors.

Therefore, the EEG components obtained in this study can be used to identify errors. Furthermore, P3, N6, and P8 clearly showed the differences between correctness and error under both static and dynamic visual stimulations, indicating that the late components were more stable to mark errors. The scalp topographical maps of N1, P2, P3, and N6 had the largest differences at the front-central electrode sites. Agreeing with this results, previous studies had shown that by analyzing the source of ErrPs, the neural generator was located at the ACC [24], and the maximum peak appeared near the electrode FCz [4,34].

### 4.2. EEG Components about the Difference between Static and Dynamic

This study was the first to focus on the difference between static and dynamic visual stimulations. Previous studies had selected static or dynamic tasks based on the purpose of the study. In this study, the difference between static and dynamic was concentrated with 300 ms after the start of stimulation. Since the difference between different stimulation modes was clear, the error recognition would be more direct and accurate. From the result of this study, dynamic N1 and P2 were lower than static ones. We speculated that the dynamic stimulus environment was more unstable than static and had more interference factors, so the dynamic EEG waveform was lower.

### 4.3. Time Window Selection under Different Visual Stimulation Modes

The differences between static and dynamic were reflected in N1 and P2 in the EEG analysis. Therefore, Tw1, tw2, tw7, and tw8 were all related to N1 or P2. In the classification of different time windows, tw3 corresponding to P3 was also statistically significant. It might be because the waveform information in the time window used for error recognition was more abundant. Although N1 showed a significant difference between correctness and error in the static case, tw1 corresponding to N1 did not get a good accuracy compared to tw2–5. However, tw6 (including tw1) achieved the highest accuracy, compared with tw7–9. Therefore, the N1 data were useful for static classification.

Combined with EEG analysis, the static condition achieved the highest accuracy with tw6, and the accuracy of the dynamic tw7 reached the highest level. Even though tw6 and tw7 did not show statistical difference under both static and dynamic conditions, there were still individual advantages. In the static case, the accuracies of 15 subjects (of 19 subjects in total) with tw6 were higher than those with tw7. In dynamic case, the accuracies of 17 subjects with tw7 were higher than those with tw6. Therefore, tw6 and tw7 related to the components sensitive to errors were still the best time window for static and dynamic stimulations, respectively.

The accuracy of tw8 in the static condition was greater than tw9, but not significantly indicating that the information of both the early and late stimuli was useful for classification under static stimulation. However, the accuracy of dynamic tw9 was significantly higher, indicating that the later stage of the dynamic stimulation was more helpful for error recognition. In general, under static stimulation, the entire piece of information deserves attention, while under dynamic one, later information was more meaningful for error recognition. Kim et al. used a dynamic cursor movement task and chose the 160–600 ms time window to observe ErrPs skipping the early information [8]. Differently, Ventouras et al. thought that only using early features classification worked best, in the static Eriksen flanker task [20]. It might be because of different task settings or feature extraction. Consistently, the error recognition of static stimulus should be focused on the features of the early stage, while the dynamic stimulus is more important in the later stage.

A limitation of this study is that the static and dynamic stimulation were not randomly arranged. The fixed sequence may lead to a sham difference between static and dynamic stimulation evoked EEG, which should be further studied in the future. Currently, we had not considered the effects of different transition times for the dynamic stimulus. The effect of lengthening or shortening the stimulation time on error recognition would be studied in the future.

## 5. Conclusions

ErrPs are expected to be used in complicated BCI systems to improve their performance. This study designed the ErrPs-based visual tasks with static and dynamic stimulations to explore the effect of different stimulations on ErrPs detection, and to help find the optimal time window with different stimulations for error recognition. The result showed that the ErrP difference between static and dynamic stimulations occurred in the early stage, while the difference between correctness and error was more reflected in the late stage. For classification, the highest accuracy was obtained at tw6 in the static condition, and at tw7 in the dynamic condition. In addition, we believed the information for the entire epoch was useful to error recognition in static tasks, and the late time window should be paid more attention to in dynamic tasks. These can reduce the ErrP-BCIs’ variability in different modal tasks and improve system performance.

## Figures and Tables

**Table 1:** Component values of correctness and error under static and dynamic visual stimulations.

Component	Time Window (ms)	Feature
N1	0–200	Negative Peak
P2	200–300	Mean
P3	300–500	Positive Peak
N6	500–800	Negative Peak
P8	800–1000	Positive Peak

**Table 2:** Component values of correctness and error under static and dynamic stimulations.

Component	Static			Dynamic		
	Correctness	Error	*p* Value	Correctness	Error	*p* Value
N1	−2.74 ± 2.30	−3.81 ± 2.20	0.0070 **	−4.94 ± 2.19	−5.51 ± 2.50	0.2122
P2	5.57 ± 2.70	5.93 ± 2.66	0.4939	1.41 ± 2.69	1.59 ± 3.02	0.4209
P3	8.76 ± 3.57	5.89 ± 3.55	0.0043 **	8.20 ± 3.17	4.93 ± 3.59	0.0010 **
N6	0.24 ± 4.37	−5.83 ± 5.37	0.0001 ***	−0.12 ± 4.35	−6.50 ± 5.27	0.0002 ***
P8	10.38 ± 8.66	14.87 ± 11.29	0.0158 *	10.38 ± 10.67	13.83 ± 12.55	0.0196 *

Values were described as the mean ± SD. *: *p* ≤ 0.05, **: *p* ≤ 0.01, ***: *p* ≤ 0.001.

**Table 3:** The difference between static and dynamic stimulations on accuracies at different time windows.

	Accuracy		
tw(i)	Static	Dynamic	Difference
tw1	58.10%	51.23%	6.87% ***
tw2	62.41%	56.70%	5.71% ***
tw3	70.53%	67.01%	3.52% **
tw4	68.50%	68.83%	−0.33%
tw5	67.44%	67.39%	0.05%
tw6	78.85%	75.81%	3.04%
tw7	77.67%	77.05%	0.62% *
tw8	75.47%	67.92%	7.55% ***
tw9	74.37%	73.77%	0.6%

*: *p* ≤ 0.05, **: *p* ≤ 0.01, ***: *p* ≤ 0.001.

**Table 4:** The difference between different time windows on accuracies at different stimulations.

		Accuracy difference (tw(i)-tw(j))	
tw(i)	tw(j)	Static	Dynamic
tw1	tw2	−4.31%	−5.47%
tw1	tw3	−12.43% ***	−15.78% ***
tw1	tw4	−10.40% ***	−17.60% ***
tw1	tw5	−9.34% **	−16.16% ***
tw2	tw3	−8.12% ***	−10.31% ***
tw2	tw4	−6.09% *	−12.13% ***
tw2	tw5	−5.03% *	−10.69% ***
tw3	tw4	2.03%	−1.82%
tw3	tw5	3.09%	−0.38%
tw4	tw5	1.06%	1.44%
tw6	tw7	1.18%	−1.24%
tw6	tw8	3.38% *	7.89% ***
tw6	tw9	4.48% **	2.04%
tw7	tw8	2.20%	9.13% ***
tw7	tw9	3.30% **	3.28% **
tw8	tw9	1.1%	−5.85% *

*: *p* ≤ 0.05, *: *p* ≤ 0.01, ***: *p* ≤ 0.001.
